# The Practitioner's Bookshelf

**Published:** 1891-10-31

**Authors:** 


### PRACTITIONERS' BOOKSHELF.

### MEDICO-LEGAL EXPERIENCE IN CALCUTTA.*

In the preface the author states that "very few original observations have been published in India, and I trust that these records will be of use to those members of the profession who are interested in the subject." Again, on page 1, he says: "It would appear that in most of our Indian Law Courts the results of Caspar's observations are assumed to furnish satisfactory data regarding post-mortem phenomena generally, that is, post-mortem phenomena are assumed to present exactly the same features whether they occur under the climatic conditions prevailing in Berlin or under those of tropical or sub-tropical regions." As this assumption of the Indian Law Courts is of course perfectly at variance with fact, the publication of these notes of medico-legal experience in India is most valuable. They relate to no less than 562 cases of medico-legal interest. First we have a chapter on the changes occurring after death, where the sequence of events shows the great effect the climate has in hastening many of those phenomena. Then follow the history of no less than eight cases of saponifiction, a large proportion to the total of cases narrated, in all of these excessive moisture and the natural heat of the climate induced the change, often within a few days of death. Three hundred and five cases of drowning are investigated and tabulated. One hundred and forty-seven cases coming under the heads of hanging, throttling, and suffocation are followed by a hundred and eleven cases of ruptures of internal organs; of these no less than thirty-four were cases of liver and twenty-nine of spleen rupture alone. The whole concludes with the narration of an interesting history of an accident in which the cause of instantaneous death was apparently the rupture of the right phrenic nerve.

The book is well written, goes straight to the point, avoiding unnecessary details, and is one of those useful pieces of work which go to make the science of legal medicine more exact. There do not seem to be any notes on medico-legal subjects in connection with the living, but all are devoted to those occurring in relation with persons found dead. It would be interesting to know in what proportion non-fatal as well as fatal assaults of all kinds are met with in Indian practice. The absence of any mention of criminal assaults on females is also noteworthy.

*By S. Corell Mackenzie, M.D., Surgeon-Major Bengal Civil Service, Professor Medical Jurisprudence Medical College of Calcutta, &c., &c. Edinburgh: E. & S. Livingstone. Pp., 100. Price 1s. 1891.

